# The effect of unusual presentation on delayed diagnosis of prostate cancer: a case series

**DOI:** 10.1186/s13256-024-04543-x

**Published:** 2024-05-22

**Authors:** Mawuenyo Attawa Oyortey, Kekeli Kodjo Adanu, Mahamudu Ayamba Ali

**Affiliations:** University of Health and Allied Sciences, Ho, Ghana

**Keywords:** Advanced prostate cancer, Orchidectomy, Unusual presentation

## Abstract

**Background:**

Early diagnosis of prostate cancer is key to achieving a cure and its proper management leads to a good prognosis. In Ghana a large percentage of patients present with advanced disease and unusual presentations in these patients result in greater delay in the diagnosis thus worsening the outcomes.

**Case presentation:**

We present three African males with advanced prostate cancer who had delayed diagnosis. The first patient, a 64 year old male presented with ascites of 2 years duration with weight loss and no lower urinary tract symptoms, the second, a 69 year old man with end stage renal failure of 6 months duration and was receiving dialysis, the third case, an 87 year old man was managed for pulmonary tuberculosis after he presented with chronic cough and lower urinary tract symptoms. All patients eventually had a prostate specific antigen done which were elevated. Further investigations including prostate biopsies, abdominopelvic CT scans for case 1, abdominopelvic ultrasound, prostate biopsies and blood urea and electrolytes for case 2, prostate biopsies, chest and lumbosacral showed a diagnosis of metastatic prostate carcinoma, and all patients were managed with androgen deprivation. The second patient received additional radiotherapy.

**Conclusion:**

A lack of knowledge of prostate cancer symptoms including unusual symptoms, can result in delayed diagnosis especially in regions of the world where a large number of patients present with advanced disease.

## Background

Early diagnosis is key for curing prostate cancer [1]. Unfortunately, approximately 33–50% of patients in a resource-constrained setting present with classical advanced disease symptoms in sub-Saharan Africa [2, 3]. This high percentage of late-stage patients may be attributed to a lack of access to or poor knowledge of early screening. In Ghana, most patients have no prior screening because of disparities in the availability of urological centres and poor knowledge of screening [4]. Asamoah *et al*. reported a 28.7% incidence of metastatic prostate carcinoma at the Korle Bu Teaching Hospital [5]. Commonly, bone pains and LUTS are the predominant symptoms at presentation in patients with prostate cancer [6, 7]. Abdominal, renal, and other distant organ metastases as the first presentation appear rare, with fewer than 50 cases reported in the literature [8]. A high index of suspicion in the assessment is therefore required for extra bony symptoms, especially in the absence of urinary symptoms. These presentations can lead to diagnostic dilemmas and delays.

We present 3 patients with advanced prostate cancer whose diagnosis was delayed.

## Case presentation

### Patient 1

A 64-year-old African man with well-controlled diabetes was referred to the urology clinic with a 2-year history of progressive abdominal distension and weight loss and was being managed for malignant ascites of unknown origin. He had undergone repeated abdominal paracentesis when the abdominal distension embarrassed his respiration. He had no lower urinary tract symptoms or family history of prostate cancer. He smoked for 7 years in the past. The physician managing him referred to the urology unit for a consideration of prostate carcinoma for the cause of his ascites after a PSA was done.

Physical examination revealed a pale and chronically ill-looking man with pitting bipedal oedema up to the mid-shin. The patient also had bilateral enlarged inguinal lymph nodes, which were hard and non-tender. There was no jaundice. The abdomen was globally distended with a positive fluid thrill. Digital rectal examination revealed a T4 prostate.

**Fig. 1:**
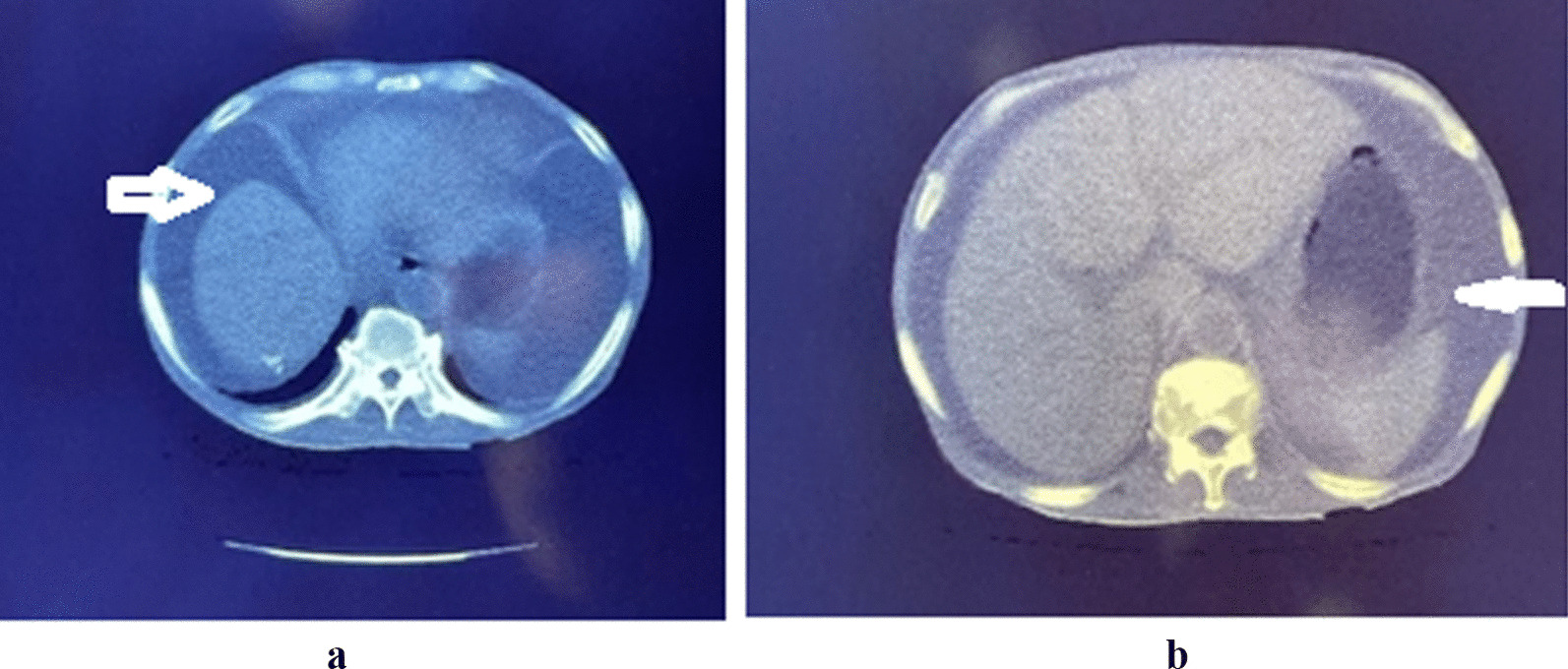
**a** Abdominopelvic computed tomography scan showing ascites (white arrow). **b** Abdominopelvic computed tomography scan showing ascites (white arrow)

The serum prostate specific antigen (PSA) concentration was 805 ng/ml, and a sextant biopsy of the prostate was performed for histopathology-diagnosed adenocarcinoma with a Gleason score of 7/10 (4 + 3) and perineural invasion. An abdominopelvic CT scan showed massive ascites with minimal bilateral pleural effusion and sclerotic vertebral bone metastases (Fig. 1a, b). The alkaline phosphatase concentration was 324 U/L (53–207). Urea concentration was 18.8 mmol/L (2.9–8.2 mmol/l), and the creatinine concentration was 190 µmol/L (62–106 µmol/l).

A diagnosis of metastatic carcinoma of the prostate with ascites and deranged renal function was made. He underwent bilateral total orchidectomy. The patient achieved complete resolution of the ascites and normalisation of renal function within a month. The patient was satisfied with his management.. His PSA dropped to 33.11 ng/ml in 3 months, 6.18 ng/ml in 8 months. He was referred to the Radiation oncologist when he became castrate resistant and was started on chemotherapy (docetaxel).

### Patient 2

A 69-year-old, African, diabetic and hypertensive patient who was not compliant with his medications was referred to our facility for hemodialysis for end-stage renal failure and was being managed by the internal medicine team. He presented with a 6-month duration of bipedal swelling and a month history of general body weakness. The patient had a prior history of urgency. He had no positive family history of prostate disease and did not smoke. Physical examination was significant for cachexia, bipedal edema up to the knee, and a Glasgow Coma Scale of 14/15. Oxygen saturation level was 98% on room air with bilateral reduction of air entry in the lower lung zones. His blood pressure was 141/79 mmHg. Digital rectal examination revealed a T4 prostate. His renal function is shown in Table 1.

**Table 1:** Renal function results before and after bilateral total orchidectomy (BTO)

Before Bilateral Total orchidectomy			After Bilateral Total orchidectomy			
Parameter	5/8/2020	9/8/2020	19/8/2020	22/8/2020	25/8/2020	2/9/2020
Creatinine (umol/l)	1870	1597	580	397	346	316
Urea (mmol/l)	18.24	59.28	33.28	34	23	19.75
Potassium (mmol/l)	4.73	4.37	3.4	3.9	3.97	3.73
Sodium (mmol/l)	149	133.4	144	149	145	132

He was initially managed for uraemic encephalopathy, and dialysis was started. An abdominal ultrasound scan showed bilateral hydronephrosis with echogenic parenchyma and a prostate volume of 81cm^3^. Patient was referred to the urologist where a PSA was done which was 11,251 ng/ml (0–4 ng/ml), and pelvic and lumbosacral X-ray images done showed osteoblastic lesions. Histology after a prostate biopsy showed adenocarcinoma with a Gleason of 4 + 4 and perineural invasion. The alkaline phosphatase activity was 124.99 U/L (53–207 U/L), and the GGT activity was 30.11 U/L (12.0–64.0 U/L). A diagnosis of obstructive uropathy secondary to carcinoma of the prostate with bone metastases was made.

The patient underwent bilateral total orchidectomy (BTO), which improved renal function; therefore, dialysis was stopped. He was also referred to a radiation oncologist to assist in his management where he had radiation to the prostate and bladder on account of the obstructive uropathy. He is currently on Abiraterone(1 g daily) and prednisolone (5 mg daily) therapy on account of castrate resistant prostate cancer and he is happy with the treatment.

### Patient 3

An 87-year-old African man who presented with a 2-week history of exacerbated chronic cough. He also experienced voiding lower urinary tract symptoms (LUTS) six months ago, which improved after treatment with 0.4 mg of tamsulosin. The cough started 6 years ago, at which time he was investigated and completed a 6-month treatment regimen for pulmonary tuberculosis. He had no family history of prostate disease and had not smoked cigarettes. He was however referred to the urologist on account of the worsening LUTS.

Physical examination revealed a chronically ill man with enlarged bilateral inguinal lymph nodes, which were hard. There were no palpable axillary lymph nodes, and he was not jaundiced. He was in respiratory distress due to the use of accessory muscles, with a respiratory rate of 28 cycles/minute and reduced air entry bilaterally. Digital rectal examination done on account of presence of LUTS and presence of inguinal lymphadenopathy revealed T4 prostate disease.

The serum prostate specific antigen (PSA) concentration was 1998 ng/ml, and sextant biopsy of the prostate was performed for histopathology. The results revealed adenocarcinoma with a Gleason score of 9/10 (5 + 4), which indicated perineural invasion and 90% glandular involvement.

There were cannon balls on chest X-ray and osteoblastic lesions on lumbosacral X-ray. The renal function test indices were normal. A diagnosis of pulmonary metastasis of prostate carcinoma was made. He underwent bilateral total orchidectomy. The patient achieved complete resolution of the chest metastasis within 2 weeks. His PSA concentration has remained low for 3 years of follow-up, and he is happy with the management. Table 2 below provides a summary of the three cases.

**Table 2:** Summary of the presented cases

	Age (yrs)	Time to diagnosis	Systems involved	Presentation	Management during the period
1	64	2 years to diagnosis	Abdominal	Ascites, weight loss	Abdominal paracentesis
2	69	6 months	Renal/bone	Renal failure	Dialysis for 1 month
3	87	3 years to diagnosis	Pulmonary/inguinal lymph nodes	Respiratory decompensation	Anti-kochs

## Discussion

In the management of men who present with signs of metastatic disease with no obvious cause, screening for prostate cancer is necessary. All the men were initially diagnosed with different pathologies and managed. However, they deteriorated within 6 months to more than 3 years. The first and the third patients presented with abdominal and pulmonary symptoms of metastatic prostate cancer for 2 and 3 years, respectively. With regards to abdominal ascites, it could result from a myriad of factors including prostate carcinoma. A high index of suspicion is required to determine the appropriate diagnosis. Both were managed for unrelated pathologies during the period before high serum prostate-specific antigen levels were detected, which prompted further investigations. The second patient, in contrast, was managed for end-stage renal failure for 6 months before diagnosis. The diagnosis of prostate cancer was eventually made in all three patients, and bilateral total orchidectomy was offered as palliative treatment. The second patient received additional radiotherapy because of bilateral ureteric obstruction.

### Epidemiology of the non-urinary presentation of prostate cancer

There have been reports in the literature of unusual presentations of prostate cancer. Presentations of intestinal obstruction; supraclavicular lymphadenopathy; and cutaneous, colonic, and omental metastases have been reported [9–11]. In a 10-year retrospective review of hospital data, Vinjamoori *et al*. reported an unusual presentation rate of 13.2% [12]. Prostate carcinoma has been found to cause pelvi-ureteric junction obstruction in a patient who experienced resolution of the obstruction after being started on LHRH analogues. The obstruction was caused by peri-ureteric obstruction from para-aortic lymph node enlargement [11].

### Clinical presentation and stages of disease

Obstructive uropathy from advanced prostate carcinoma has been duly reported in the literature. It is important to have a high index of suspicion of prostate cancer in elderly men presenting with end-stage renal disease. An abdominopelvic ultrasound, which is readily available in most parts of the world, should be requested to rule out obstructive uropathy for appropriate management. This approach will reduce the cost to patients who would otherwise be on dialysis, which is expensive and not covered by health insurance in Ghana. Obstructive uropathy can be due to obstruction at the bladder neck or from obstruction of the ureteric orifices [13]. The earlier the diagnosis and definitive management are, the better the return to normal renal function will be. This patient had some residual renal damage but was not dialysis dependent after androgen deprivation and radiotherapy treatment. Obstructive uropathy has been associated with significantly worse overall survival than patients without obstructive uropathy [13].

A lack of knowledge of prostate cancer and its symptoms contributes to delayed presentation and diagnostic delays by healthcare providers [3]. According to the World Health Organisation (WHO) guidelines for early cancer detection, the importance of creating awareness and improving access to healthcare as well as providing early clinical diagnosis, staging and early referral for treatment is key to tackling the increasing burden of prostate cancer, especially in our part of the world [14].

### Management options

There are several treatment options for the management of advanced or metastatic prostate cancer. Androgen deprivation therapy plays a major role in these patients, and this process can be performed medically or surgically [15]. In our part of the world, cost to the patient plays a major role, as treatment for prostate cancer is not covered by national health insurance [16]. Several studies have compared intermittent androgen deprivation to continuous androgen deprivation when the medical route is chosen. Newer generation antiandrogens and chemotherapies, such as docetaxel, have also been used [17]. The treatment option chosen is patient centred, and the patient decision after appropriate counselling should be considered.

### Follow-up prognosis after metastatic presentation

Follow-up and supportive treatment after the diagnosis of advanced prostate cancer are important management steps, as patients can develop castration resistance at some point during treatment and may need to be treated with second-line antiandrogens [17, 18]. These procedures are usually quite expensive and inaccessible financially for some patients, especially those in low-income countries, and thus, most of these procedures are managed palliatively [16].

## Conclusions

In elderly men, unusual signs and symptoms or treatment failure for medical conditions may be the only early indicator of prostate disease for which clinical and diagnostic investigations must be commenced. This case series highlights the importance of having a high index of suspicion in men presenting with unusual symptoms of metastatic disease and renal failure. A good clinical history, digital rectal examination and serum prostate-specific antigen analysis are important for investigating prostate malignancy.

## Data Availability

All available data are included in the text, tables and figures of the manuscript.

## Abbreviations

PSA: Prostate Specific Antigen
BTO: Bilateral Total orchidectomy
LUTS: Lower urinary tract symptoms
WHO: World Health Organization
